# Preoperative Y-90 microsphere selective internal radiation treatment for tumor downsizing and future liver remnant recruitment: a novel approach to improving the safety of major hepatic resections

**DOI:** 10.1186/1477-7819-7-6

**Published:** 2009-01-08

**Authors:** Seza A Gulec, Kenneth Pennington, Michael Hall, Yuman Fong

**Affiliations:** 1 Center for Cancer Care at Goshen Health System, Goshen, IN, USA; 2 Memorial Sloan-Kettering Cancer Center, New York, NY, USA; 3 Current address : Florida International University, College of Medicine, Miami, FL 33199, USA

## Abstract

**Background:**

Extended liver resections are being performed more liberally than ever. The extent of resection of liver metastases, however, is restricted by the volume of the future liver remnant (FLR). An intervention that would both accomplish tumor control and induce compensatory hypertrophy, with good patient tolerability, could improve clinical outcomes.

**Case presentation:**

A 53-year-old woman with a history of cervical cancer presented with a large liver mass. Subsequent biopsy indicated poorly differentiated carcinoma with necrosis suggestive of squamous cell origin. A decision was made to proceed with pre-operative chemotherapy and Y-90 microsphere SIRT with the intent to obtain systemic control over the disease, downsize the hepatic lesion, and improve the FLR. A surgical exploration was performed six months after the first SIRT (three months after the second). There was no extrahepatic disease. The tumor was found to be significantly decreased in size with central and peripheral scarring. The left lobe was satisfactorily hypertrophied. A formal right hepatic lobectomy was performed with macroscopic negative margins.

**Conclusion:**

Selective internal radiation treatment (SIRT) with yttrium-90 (Y-90) microspheres has emerged as an effective liver-directed therapy with a favorable therapeutic ratio. We present this case report to suggest that the portal vein radiation dose can be substantially increased with the intent of inducing portal/periportal fibrosis. Such a therapeutic manipulation in lobar Y-90 microsphere treatment could accomplish the end points of PVE with avoidance of the concern regarding tumor progression.

## Background

Extended liver resections, with an operative mortality of less than 5%, are being performed more liberally than ever. This has come about as a result of advances in surgical, anesthetic and perioperative care, along with improvements in medical imaging that have allowed better patient selection and surgical planning. At many centers, more than two-thirds of liver resections now consist of major hepatectomies. Liver resection has also been recognized as the only treatment that offers meaningful improvement in survival in patients with colorectal cancer liver metastases (CRCLMs). Indications for surgical resection continue to expand. An increasing number of patients with hepatocellular carcinoma (HCC), neuro-endocrine tumor metastases, and, more selectively, patients with other metastatic cancers are being considered for surgical treatment [1].

The extent of resection of liver metastases is restricted by the volume of the future liver remnant (FLR). Among different strategies, portal vein embolization (PVE) has gained wider acceptance to achieve the goal of increasing the volume of the FLR [2]. First reported by Makuuchi et al. [3], the aim of PVE is to bring about atrophy of the segments to be resected and induce a compensatory hypertrophy of the remaining segments [4]. This technique was first applied to patients with Klatskin tumors, and its indications have been subsequently extended to patients with metastatic liver tumors [3-9]. Induction of hypertrophy of the nondiseased portion of the liver reduces the risk of hepatic insufficiency and associated complications after resection. Clinically adequate compensatory hypertrophy occurs approximately 2 to 3 weeks postinduction [10]. An FLR of > 20% in patients with an otherwise normal liver, > 30% for those who have received extensive chemotherapy, and > 40% in patients with hepatic fibrosis/cirrhosis is recommended for a safe major hepatic resection [2,10]. A recent meta-analysis concluded that PVE is a safe and effective procedure for inducing liver hypertrophy to prevent postresection liver failure due to insufficient liver remnant [11]. The controversy over the possibility of tumor progression in nonembolized (and also in embolized) segments during the induction period, however, remains unresolved. An intervention that would both accomplish tumor control and induce compensatory hypertrophy, with good patient tolerability, could improve clinical outcomes.

Selective internal radiation treatment (SIRT) with yttrium-90 (Y-90) microspheres has emerged as an effective liver-directed therapy with a favorable therapeutic ratio. SIRT, both as a stand-alone therapy and in conjunction with systemic or regional chemotherapy (chemo-SIRT), has been demonstrated to be an effective modality in the management of primary and metastatic liver tumors [12-16]. Y-90 microspheres, injected via the hepatic artery, are entrapped within the tumor (preferentially) and hepatic arterial microvasculature, and emit high-energy β radiation. The high tumor-to-liver concentration ratio, along with the short range of β particles limiting radiation damage to the hepatocellular parenchyma, result in relatively safe delivery of tumoricidal radiation doses to the tumors [17]. Sophisticated dosimetric techniques allow estimation of the tumor and liver radiation doses [18].

We suggest that the portal vein radiation dose can be substantially increased with the intent of inducing portal/periportal fibrosis. Such a therapeutic manipulation in lobar Y-90 microsphere treatment could accomplish the end points of PVE with avoidance of the concern regarding tumor progression.

## Case presentation

**Figure 1:**
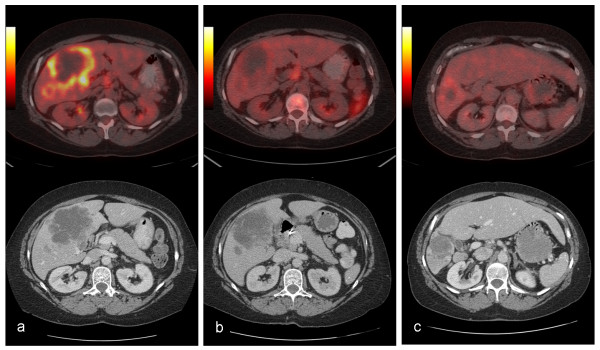
**FDG-PET/CT image sets demonstrating progressive decrease in the functional and anatomic volume of the tumor with concurrent left lobe hypertrophy**. a: Pre-treatment, b: 4-weeks after first SIRT treatment, c: At the completion of the full course of the treatment.

**Figure 2:**
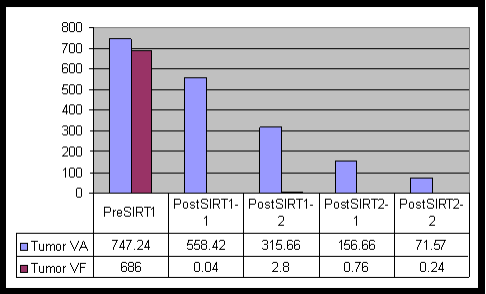
**Graph analysis of anatomic (VA) and functional (VF) tumor volume changes during the course of the treatment**. Functional volume following the first course of the treatment becomes too low to be depicted on this graph.

**Figure 3:**
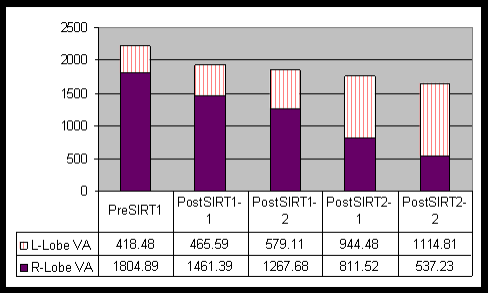
**Graph analysis of left and right hepatic lobe volumes during the course of the treatment**. Note the progressive increase in left lobe volume with concurrent decrease in the right lobe volume.

A 53-year-old woman with a history of cervical cancer presented with a large liver mass. Subsequent biopsy indicated poorly differentiated carcinoma with necrosis suggestive of squamous cell origin. Six years earlier, this patient had been diagnosed with locally advanced cervical cancer. At that time, she received chemoradiation and achieved a complete clinical response with no detectable residual tumor at the completion of treatment. The current imaging assessment with fluorodeoxyglucose (FDG) positron emission tomography (PET)/computed tomography (CT) indicated a large necrotic tumor occupying the greater part of the right lobe (Figure 1a). This patient was thought to be a reasonable candidate for surgical resection based on her relatively long disease-free interval and the absence of any locoregional recurrence or detectable extrahepatic disease. A decision was made to proceed with pre-operative chemotherapy and Y-90 microsphere SIRT with the intent to obtain systemic control over the disease, downsize the hepatic lesion, and improve the FLR. Hepatic angiography demonstrated that the right hepatic artery (arising from the celiac axis and supplying segments 4–8) provided the entirety of the liver mass. The gastroduodenal and right gastric arteries were coil embolized to prevent gastrointestinal (GI) reflux during microsphere administration. Hepatic arterial technetium 99 m (^99 m^TC) macroaggregated albumin (MAA) liver scan confirmed the absence of extrahepatic GI and pulmonary uptake. Next, medical internal radiation dosimetry (MIRD) was used to determine projected tumor and liver absorbed doses. Y-90 resin microspheres (SIR-Spheres, (SIRTeX Medical Limited, North Ryde, Australia) were administered to the right hepatic lobe 24 hours after initiation of systemic chemotherapy with 5-fluorouracil (5-FU)-leucovorin-oxaliplatin (FOLFOX-6). Microspheres were instilled directly into the right hepatic artery via a 3 french microcatheter. A maximum safe *administered dose *was given (2.7 GBq, determined per fluoroscopic criteria) with estimated tumor and liver *absorbed doses *of 90 Gy and 30 Gy, respectively. The treatment was well tolerated with no untoward effects. A 4-week post-treatment FDG-PET/CT scan demonstrated a complete metabolic response, and a 25% decrease in anatomic volume (Figure 1b, 2). Liver function tests remained normal except for a mild elevation in alkaline phosphatase level. The patient's physical performance status showed remarkable improvement over the next 3 months, and serial PET/CT imaging studies indicated further decrease in anatomic volume (Figure 2). Metabolic status of the tumor remained depressed (Figure 2). The left lobe volume showed progressive increase (Figure 3). The patient continued systemic chemotherapy for a total of 4 courses with no significant toxicity noted. At 3-month follow-up evaluation, based on a favorable tumor response, the patient's improved performance status, and the absence of any evidence of extrahepatic disease progression, a decision for a second course of SIRT (without chemotherapy) was made. A second maximum safe administered dose of Y-90 resin microspheres was given (1.3 GBq, determined per fluoroscopic criteria) in the right hepatic lobe with estimated tumor and liver absorbed doses of 80 Gy and 25 Gy, respectively. This second course was also well tolerated, with no early or late complications. Three months after the second SIRT, tumor anatomic volume decreased to 10% of the pretreatment size (Figures 1c, 2). No increase in metabolic activity was observed. Left liver lobe volume showed 2.7 times increase from the pretreatment value (Figures 1c, 3). Liver function tests remained normal. A transient increase in splenic volume was observed during the follow-up; however, no significant volume difference was noted between the pre-SIRT and 3-month post-SIRT splenic volumes.

**Figure 4:**
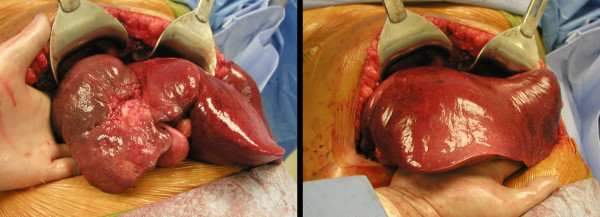
**Intra-operative pictures demonstrating significantly down-sized tumor with scarring and, major left lobe hypertrophy**.

A surgical exploration was performed six months after the first SIRT (three months after the second). There was no extrahepatic disease. The tumor was found to be significantly decreased in size with central and peripheral scarring. The left lobe was satisfactorily hypertrophied (Figure 4). No evidence to indicate portal hypertension was noted. A formal right hepatic lobectomy was performed with macroscopic negative margins. Postsurgical course was uneventful. The patient was discharged on the fourth postoperative day.

**Figure 5:**
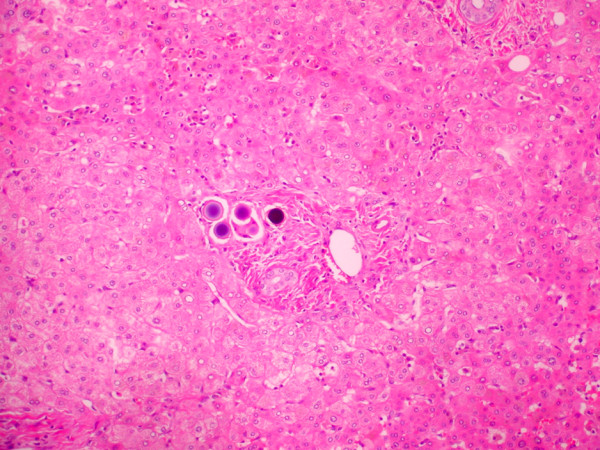
**Surgical pathology findings**. Tumor necrosis and degeneration with microspheres in the treatment field (10×).

Surgical pathology indicated microsphere localization within the tumor producing more than 90% pathologic response. The surrounding liver tissue showed portal triaditis with mononuclear inflammatory cellular response predomination and portal and periportal fibrosis (Figure 5). Hepatocellular architecture/morphology demonstrated a mild centriacinar steatosis. No inflammatory/fibrotic changes were observed in the central vein region. Reticulin stain demonstrated intense fibrotic reaction in the portal tracts.

## Discussion

Tumor progression in nonembolized liver segments during the hypertrophy induction period is more than a theoretical concern. A number of reports have shown that the volume of metastatic liver tumors increased more rapidly compared with the volume of the liver parenchyma after PVE, both in nonembolized and embolized liver segments [19-21]. Hemming et al reported intra- or extrahepatic disease progression in 5 of 39 patients in the post-PVE period, which raised the concern that PVE may actually be promoting tumor growth via induction of growth factor/cytokine release [22]. In a more recent study, Hayashi et al. demonstrated that liver tumor growth in embolized lobes accelerated after PVE in patients with HCC [23].

Trans-arterial Y-90 microspheres are a viable treatment option for unresectable liver tumors. Y-90 microspheres always localize in the portal tracts. The deposition of the majority of the absorbed dose is within a very tight zone immediately surrounding the microspheres. Even though the maximum range of β particles in the liver is slightly greater than 10 mm, more than 90% of the absorbed dose is deposited within the portal tract at a distance within 30 μm from the microspheres [unpublished data]. The clinical translation of this result is that the greatest absorbed dose effect is exerted on the portal triad structures. An interlobular portal vein branch, at a distance of 50 μm from a microsphere (microsphere cluster), could receive twice the average liver dose calculated by standard MIRD technique.

Gray et al. have indicated that SIRT could be associated with subclinical portal hypertension [24]. These authors noted a significant increase in portal vein diameter and spleen volume by 12 months after treatment. The increase in spleen volume and portal vein size was thought to be due to portal hypertension resulting from scarring within the liver as a result of radiation effect. Histopathologic review of SIRT-treated liver specimens reveals portal/periportal fibrosis. This is best illustrated with trichrome (Mason) staining, as was also demonstrated in our case. The periportal fibrosis could result in a decrease in the flow to the hepatocellular parenchyma, which potentially could initiate similar physiologic responses induced with PVE, leading to contralateral lobe hypertrophy.

Obviously, the major advantage of SIRT is the effective control of the tumor in the target liver lobe. The value of initiating chemotherapy concomitantly with Y-90 microsphere administration not only serves the objective of radiosensitization, but also accomplishes the need/benefit of tumor suppression at a systemic level. Fong et al. have recently demonstrated that chemotherapy could minimize/eliminate the risk of tumor growth, which otherwise could be problematic if PVE was performed without a systemic coverage [unpublished data].

## Conclusion

Y-90 microsphere SIRT/chemo-SIRT effectively controls tumor growth. With appropriate scaling of radiation absorbed dose to the lobar portal microvascular bed, it also could induce contralateral lobe hypertrophy. The simultaneous accomplishment of tumor control and FLR recruitment might offer a better therapeutic profile compared with that of PVE. Clinical indications, patient selection criteria, and dosimetry for this therapeutic manipulation require further investigation.

## Consent

Written informed consent was obtained from the patient for publication of this case report.

## Competing interests

The authors declare that they have no competing interests.

## Authors' contributions

SG is the authorized user for Y-90 microsphere administration and performed the hepatic resection. MH is the interventional radiologist who performed the Y-90 microsphere treatment. KP is the medical oncologist. SG, MH, and YF co-wrote the case report.
